# Polysaccharide Peptide from *Ganoderma lucidum* Reduces Acute Kidney Injury Through Regulating the Integrin β3/Fn1 Axis

**DOI:** 10.3390/biom16040610

**Published:** 2026-04-20

**Authors:** Hatungimana Mediatrice, Hongjian Luo, Lianfu Wang, Yang Yao, Zhujun Liu, Nsanzinshuti Aimable, Yingping Hu, Yukun Zhang, Zhanxi Lin, Dongmei Lin

**Affiliations:** 1 National Engineering Research Center of JUNCAO Technology, College of Life Science, Fujian Agriculture and Forestry University, Fuzhou 350002, China; mediatunga@gmail.com (H.M.); fafuer2025@fafu.edu.cn (H.L.); 000xm19024@fafu.edu.cn (L.W.); nsanziaima@gmail.com (N.A.); yingpinghu33@gmail.com (Y.H.); 000q121034@fafu.edu.cn (Z.L.); 2 Rwanda Agriculture and Animal Resources Development Board, Kigali P.O. Box 5016, Rwanda; 3 Cross-Strait Cooperation Base on Agricultural Science and Technology Industry (Membership) Under the Ministry of Science and Technology (MOST), College of Agriculture, Fujian Agriculture and Forestry University, Fuzhou 350002, China; 4 Chongqing Key Laboratory of Development and Utilization of Genuine Medicinal Materials in Three Gorges Reservoir Area, Chongqing 404120, China; yangyao@cqtgmc.edu.cn (Y.Y.); 20250130@cqtgmc.edu.cn (Z.L.); 5 State Key Laboratory of Natural and Biomimetic Drugs, Department of Pharmacology, School of Basic Medical Sciences, Peking University, Beijing 100191, China

**Keywords:** acute kidney injury, *Ganoderma lucidum*, polysaccharide peptides, ischemia–reperfusin, integrin β3/fibronectin axis

## Abstract

Acute kidney injury (AKI) continues to pose a significant clinical challenge due to its high morbidity rates and limited therapeutic options. Recent evidence suggests that natural compounds may provide renoprotective benefits by modulating oxidative stress and inflammation. This study examines the protective effects of a novel polysaccharide peptide extracted from Ganoderma lucidum (GL-PPQ1) against renal ischemia–reperfusion (I/R) injury, with particular emphasis on the integrin β3/Fibronectin 1 (Fn1) signaling axis. A murine model of renal I/R injury was established, and GL-PPQ1 was administered orally for seven days before surgery. The assessment included renal function, histopathology, oxidative stress markers, and inflammatory cytokines. Additionally, transcriptomic profiling and protein expression analyses were conducted to elucidate the underlying mechanisms. The results revealed that GL-PPQ1 pretreatment significantly reduced renal tubular damage, lowered serum creatinine and blood urea nitrogen levels, and diminished oxidative stress and inflammatory responses. RNA sequencing revealed that GL-PPQ1 affected gene sets associated with extracellular matrix remodeling and cell adhesion. Western blot and immunohistochemistry further confirmed that GL-PPQ1 decreased the expression of integrin β3 and Fn1, suggesting a regulatory effect on their interaction during I/R injury. These findings demonstrate that GL-PPQ1 offers substantial kidney protection by mitigating oxidative stress, inflammation, and dysregulation of the integrin β3/Fn1 signaling pathway. Thus, this study supports that polysaccharide peptides derived from Ganoderma lucidum could have the potential to serve as both a dietary supplement and a therapeutic agent in the treatment of AKI.

## 1. Introduction

Acute kidney injury (AKI) is a prevalent and critical clinical condition defined by a swift decline in renal function, frequently linked to significant morbidity and mortality [1]. The etiology of AKI is multifactorial, encompassing a range of mechanisms including ischemia–reperfusion injury, sepsis, nephrotoxic agents, and inflammatory responses [2,3]. This condition necessitates prompt recognition and intervention to mitigate its implications on patient outcomes. Despite advances in supportive care, effective pharmacological treatments for AKI remain limited. Therefore, identifying novel therapeutic agents with renal protective properties is of critical importance [4].

*Ganoderma lucidum* (*G. lucidum*) is a traditional medicinal mushroom widely used in East Asian countries for its diverse pharmacological properties. Among its bioactive constituents, polysaccharide peptides (GLPPs) have attracted increasing attention due to their potent anti-inflammatory, antioxidant, immunomodulatory, and organ-protective effects [5,6]. Previous studies have demonstrated that GLPP, extracted from the fruiting body of *G. lucidum* via membrane separation (10 kDa cut off), exhibits significant protective effects against renal ischemia–reperfusion injury, hyperuricemia, and proteinuric nephropathy by modulating oxidative stress, inflammatory cytokines, and renal urate transporters [7,8,9]. From further refinement of GLPP, two more homogeneous components, GL-PPSQ2 and GL-PPQ3, were isolated from GLPP through sequential 40–66% ethanol precipitation and gel-based preparative liquid chromatography. The former showed enhanced bioactivity in inhibiting neutrophil extracellular trap (NET) formation and alleviating intestinal and pulmonary injuries in ischemia–reperfusion models. Structurally, GL-PPSQ2 is characterized by a highly branched polysaccharide backbone with O-glycosidically linked peptides, and its molecular weight and monosaccharide composition have been precisely defined [10]. Importantly, GL-PPQ3 (41.08 kDa) significantly exhibited gastroprotective activity against ethanol-induced ulcers by promoting mucosal defense, mitigating oxidative stress and apoptosis, via modulation of the FAK/MAPK pathway and NLRP3 inflammasome [11]. These findings underscore the therapeutic potential of structurally defined GLPP derivatives in managing acute organ injuries.

Building upon this foundation, a novel GLPP derivative, designated GL-PPQ1, was obtained via membrane separation (10 kDa cutoff) and 40% ethanol precipitation. This modification in ethanol concentration during purification may influence the polysaccharide–peptide ratio, molecular weight distribution, and branching pattern, potentially altering its pharmacological profile. Preliminary studies suggest that GL-PPQ1 retains strong renoprotective activity, and we hypothesize that it may attenuate acute kidney injury (AKI) via a novel mechanism. Integrins, particularly integrin β3, are transmembrane receptors that mediate cell–extracellular matrix (ECM) interactions and play pivotal roles in tissue repair and inflammation [12,13]. Fibronectin 1 (Fn1), an essential ECM glycoprotein, is known to interact with integrin β3 and influence cell adhesion, migration, and signaling during tissue injury and recovery processes. Based on RNA sequencing, dysregulation of the Fn1 and integrin axis by GL-PPQ1 may play an important role in AKI, as previously reported by Hassan HM et al. and Halper (2021) [14,15].

In this study, we investigate the renoprotective effects of a novel *G. lucidum*-derived polysaccharide peptide, GL-PPQ1, in a murine model of AKI. We particularly focus on its role in modulating the integrin β3/Fn1 interaction, aiming to elucidate a novel mechanism by which these natural compounds exert therapeutic benefits. Our findings provide new insights into the development of structure-optimized GLPP analogs as targeted, natural dietary supplements and therapeutic agents for the prevention and treatment of AKI.

## 2. Materials and Methods

### 2.1. Preparation of Ganoderma lucidum Polysaccharide Peptide

**Figure 1:**
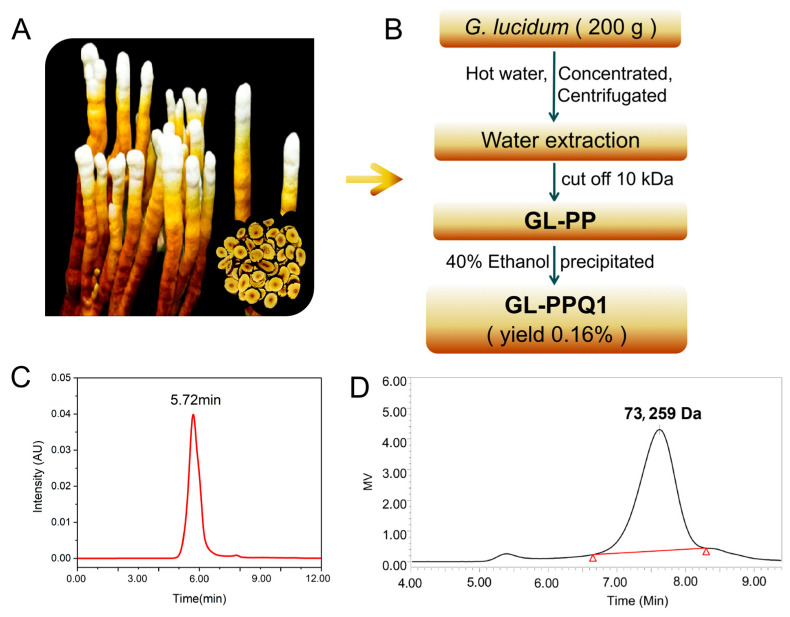
Isolation, purity, and molecular weight characterization of GL-PPQ1 from antler-shaped *G. lucidum*. (**A**) Morphology of the fruiting body. (**B**) Schematic flowchart for polysaccharide-peptide GL-PPQ1 preparation. (**C**) HPLC chromatogram for purity analysis. (**D**) HPGPC profile for molecular weight determination. Triangles are used as peak markers.

Antler-shaped *Ganoderma lucidum* fruit bodies (Figure 1A) were obtained from the National Engineering Research Center of JUNCAO Technology. A total of 200 g of dried fruiting body of *G. lucidum* was extracted twice with 3 L of hot distilled water for 2 h, followed by centrifugation at 10,000 r/min for 10 min. The concentration and separation were performed via the AKTA Flux 6 hollow fiber membrane system (AKTAflux6, GE, Marietta, GA, USA) with a 10 kDa column (10 kDa up retentate and permeate. With some modifications to the previous protocols [11,16], the current concentrate was size-fractionated through a 10 kDa membrane. The retentate was gathered and concentrated, followed by precipitation with 40% (*v/v*) ethanol. The retentate was gathered and concentrated, followed by precipitation with three volumes of ethanol (*v*/*v*). This resulting precipitate was then collected and stored at 4 °C for 12 h. The precipitate was centrifuged at 10,000 r/min for 10 min at 4 °C and dried in a vacuum freeze-drier, designated as GL-PP. The GL-PP was then redissolved in distilled water and precipitated with 40% (*v/v*) ethanol. Following centrifugation and lyophilization, the resulting precipitate was obtained as GL-PPQ1 (Figure 1B).

### 2.2. Experimental Animals

Male C57BL/6N mice (6–8 weeks old, weighing 20–23 g) were sourced from the Laboratory Animal Center at Peking University (Beijing, China). The sample size of *N* = 6 per group was determined based on previously published renal I/R injury studies using similar murine models [17], which demonstrated adequate statistical power to detect significant differences in renal function parameters. Mice were randomly allocated to experimental groups using a simple random number assignment method before treatment initiation. The method used complied with the ARRIVE guidelines and the National Research Council’s recommendations regarding the Care and Use of Laboratory Animals. Furthermore, all protocols were granted the necessary approval from the Institutional Animal Care and Use Committee at Peking University Health Science Center on 17 February 2023, under approval number BCJB0025, ensuring compliance with ethical standards in animal research.

### 2.3. Induction of the Kidney Ischemia–Reperfusion Injury in Mice Model

The mice had unrestricted access to food and water and were maintained under a 12 h light/dark cycle to ensure a stable physiological environment. One week before surgical intervention, the mice received daily oral administration of GL-PPQ1 at a dose of 100 mg/kg for a consecutive 7-day period. To induce renal ischemia–reperfusion (I/R) injury, the animals were anesthetized using pentobarbital (50 mg/kg), and the experimental renal I/R model was established under controlled conditions as described by previous researchers. Sham-operated animals underwent the same surgical procedure but without arterial clamping. After surgery, all mice had unrestricted access to water and chow. The ischemic phase lasted 40 min, followed by a 6 h reperfusion period. At the end of the study, mice were euthanized via an overdose of anesthesia, and the kidney tissues were harvested for further analysis. All euthanasia procedures were conducted under pentobarbitone anesthesia, with rigorous measures taken to minimize animal distress [18,19].

### 2.4. Histopathological Examination

The kidneys of mice were washed with saline and promptly fixed in 10% neutral buffered formalin for 24–72 h. Subsequently, they were embedded in paraffin, sectioned, and stained with Hematoxylin and Eosin (H&E). Blinded observers examined kidney sections using light microscopy, and injury was evaluated. The slides were subsequently assessed using a semi-quantitative scoring method. Kidney lesions were evaluated based on the observed pathological changes associated with the I/R model. These changes included cell necrosis, formation of intratubular casts, reduction of the brush border, tubular dilation, and tubule regeneration. Scoring was conducted on nine randomly selected fields of view from the outer stripe of the outer medulla and the cortex [20].

Kidney injury scoring was based on the presence of proximal tubular brush border, tubular epithelial swelling and vacuolation, tubular necrosis/cast formation, intratubular cast formation, and interstitial edema and inflammatory cell infiltration. Each parameter was scored on a four-level scale: 0 (absent), 1 (mild), 2 (moderate), and 3 (severe). The final score was obtained by averaging the individual assessment scores across all criteria [21].

### 2.5. Analysis of Serum Biochemical Indexes

To quantify the severity of ischemia–reperfusion (I/R) injury, we measured key oxidative stress and cellular damage markers, including superoxide dismutase (SOD), glutathione (GSH), lactate dehydrogenase (LDH), and malondialdehyde (MDA) in serum and tissue samples. These biomarkers were analyzed using commercial colorimetric assay kits (Nanjing Jiancheng Bioengineering, Nanjing, China) per the manufacturer’s instructions.

### 2.6. Quantification of Inflammatory and Kidney Injury Markers by ELISA

Blood samples were centrifuged at 3000 r/min for 15 min to obtain serum. Serum levels of myeloperoxidase (MPO) and pro-inflammatory cytokines, including tumor necrosis factor-α (TNF-α), interleukin-1β (IL-1β), and interleukin-6 (IL-6) were quantified using commercial enzyme-linked immunoassay (ELISA) kits (Jiangsu Meibiao Biotechnology Co., Ltd., Nanjing, Jiangsu, China) according to the manufacturer’s protocols.

### 2.7. Assessment of Apoptosis by TUNEL Assay

Apoptotic cells in paraffin-embedded kidney tissue sections were detected using a commercial TdT-mediated dUTP Nick-End Labeling (TUNEL) assay kit (Elabscience, Shanghai, China), following established protocols. Nuclei exhibiting DNA fragmentation were identified by distinct green fluorescence staining, which served as the indicator of TUNEL-positive apoptotic cells [22].

### 2.8. Cell Culture

HK-2 cell cultures (Haixing Biosciences, Suzhou, China) were maintained in DMEM/F-12 medium (Haixing Biosciences, Suzhou, China) supplemented with 10% fetal bovine serum (FBS) (Haixing Biosciences, Suzhou, China) in a 37 °C, 5% CO_2_ incubator. The cells were exposed to GL-PPQ1 (0–1280 mg/L) for 24 h, and cell viability was assessed using the CCK-8 assay. The sample size *N* = 3 independent biological replicates, each derived from separate cell culture experiments. Results indicated that a concentration of 160 mg/L GL-PPQ1 reduced cell viability to below 50%, which was defined as sufficient cytotoxic damage based on widely accepted in vitro toxicology criteria [23].

### 2.9. OGD/R Cell Model

To simulate ischemia–reperfusion injury in vitro, HK-2 cells were subjected to oxygen-glucose deprivation/reoxygenation (OGD/R). Cells were washed with phosphate-buffered saline (PBS) and incubated in glucose-free DMEM under hypoxic conditions (1% O_2_, 5% CO_2_, 94% N_2_) at 37 °C for 6 h. Following OGD, cells were returned to normal culture conditions (DMEM/F-12 with 10% FBS, 37 °C, 5% CO_2_) for 12 h of reoxygenation [24]. Control cells were maintained under standard normoxic conditions throughout. The 6 h deprivation and 12 h reoxygenation periods were selected based on established protocols that reliably reproduce ischemia-like cellular injury, including oxidative stress and apoptosis, without causing complete cell death, thereby allowing assessment of cytoprotective interventions [25]. Compared to the in vivo I/R model (40 min ischemia, 6 h reperfusion), the OGD/R model provides a complementary and mechanistically consistent platform for evaluating the direct cellular effects of GL-PPQ1 under controlled conditions.

### 2.10. Cell Viability Detection

Cell viability was assessed using CCK-8 kits (Biosharp, Beijing, China) according to a previous study. The cell viability of HK-2 cells was calculated using the following formula: (absorbance of sample well—absorbance of blank well)/(absorbance of control well—absorbance of blank well) × 100%.

### 2.11. ROS Assay

HK-2 cells were seeded in 6-well plates and treated as experimental groups. After treatment, cells were incubated with 10 μM DCFH-DA (ROS probe, dissolved in serum-free medium) at 37 °C for 20 min in the dark. Then, cells were washed twice with pre-cooled PBS to remove unloaded probe, trypsinized, and resuspended in 500 μL PBS. Reactive oxygen species (ROS) levels were detected using a flow cytometer (BD Accuri C6). Green fluorescence (DCF, excitation: 488 nm; emission: 525 nm) of 10,000 cells per sample was collected. Data were analyzed with FlowJo software v10.8.1, and ROS production was expressed as the mean fluorescence intensity (MFI) relative to the control group.

### 2.12. Apoptosis Detection

HK-2 cells were treated as experimental groups, then harvested by trypsinization (without EDTA), and washed twice with cold PBS. Cells were resuspended in 500 μL PBS, and 5 μL Hoechst 33,342 (100 μg/mL) and 5 μL PI (50 μg/mL) were added, and then incubated at 37 °C for 15 min in the dark. Samples were analyzed using a flow cytometer (BD Accuri C6): Excite Hoechst at 350 nm (emission: 460 nm, blue fluorescence) and PI at 488 nm (emission: 610 nm, red fluorescence). In total, 10,000 events per sample were collected. Viable cells (Hoechst^+^/PI^−^), early apoptotic cells (Hoechst^++^/PI^−^), and late apoptotic/necrotic cells (Hoechst^++^/PI^+^) were gated via FlowJo software.

### 2.13. Western Blot

The protein extracted from the kidney tissues was quantified with bicinchoninic acid reagent (Epizyme Biotech, Shanghai, China). The same amount of protein was separated by SDS-PAGE (Bioman, Beijing, China), and then the separated protein was transferred to the polyvinylidene difluoride (PVDF) membranes. The membranes were incubated with primary antibody for 12 h at 4 °C. After sealing in skimmed milk for 1 h at 20 °C, the membranes were probed with horseradish peroxidase-labeled secondary antibody at 20 °C for 2 h. The protein bands were visualized using super-sensitive enhanced chemiluminescence (ECL) luminescence reagent (Meilunbio, Dalian, China) in the dark, and then use qTouch Western Blot Imager (RWD Life Science Co., Ltd., Shenzhen, China) for imaging. The densitometric analysis was performed by Image J software v6.1.

### 2.14. Immunohistochemistry

Renal tissues were fixed in 4% paraformaldehyde, dehydrated, and embedded in paraffin [26]. Sections (4 μm) were cut, mounted on poly-L-lysine slides, and underwent deparaffinization and rehydration. Antigen retrieval was performed by heating in citrate buffer (pH 6.0) at 95 °C for 15 min. Endogenous peroxidase activity was quenched with 3% hydrogen peroxide, followed by blocking with 5% BSA [27]. Primary antibodies (anti-integrin β3, anti-fibronectin, and anti-KIM-1) were incubated overnight at 4 °C. After washing, sections were treated with HRP-conjugated secondary antibodies, and signals were developed using 3,3′-Diaminobenzidine (DAB), followed by hematoxylin counterstaining [28].

### 2.15. RNA Sequencing and Bioinformatics Analysis

Total RNA was extracted from kidney tissues of sham, I/R, and I/R + GL-PPQ1-treated mice and subjected to transcriptomic profiling by RNA sequencing. Raw sequencing reads were processed and aligned to the mouse reference genome using standard bioinformatics pipelines. Differentially expressed genes (DEGs) were identified using a threshold of |log2 fold change| ≥ 1 and an adjusted *p*-value < 0.05. False discovery rate (FDR) correction was applied using the Benjamini–Hochberg method to account for multiple testing and minimize the risk of false-positive discoveries. Gene Ontology (GO) enrichment analysis was performed to identify significantly enriched biological processes and molecular functions among DEGs. Protein–protein interaction (PPI) network analysis was conducted using STRING to explore functional relationships among overlapping DEGs across comparison groups.

### 2.16. Statistical Analysis

The collected data were subjected to analysis using the Statistical Package for the Social Sciences v18.0 (SPSS) and visualized by GraphPad 8.0. These data were expressed as mean ± standard deviation. Before statistical analysis, the normality of data distribution was formally assessed using the Shapiro–Wilk test, which confirmed that all datasets were approximately normally distributed (*p* > 0.05), supporting the use of parametric statistical methods. One-way Analysis of Variance (ANOVA) was employed for the overall comparisons among treatments, with subsequent pairwise comparisons conducted using the least significant difference (LSD) test. *p* < 0.05 was considered statistically significant [29]. A *p*-value of less than 0.05 was considered statistically significant.

## 3. Results

### 3.1. Homogeneity and Molecular Weight Determination

The polysaccharide-peptide GL-PPQ1 was isolated from *G. lucidum* (Figure 1A). The extraction and purification process of GL-PPQ1 is shown in Figure 1B. The yield of GL-PPQ1 is 0.16% based on the dry weight of the fruiting bodies. High-performance gel permeation chromatography (HPGPC) analysis combined with peak area normalization indicated a high purity of 98.82%, and a polydispersity index of 1.19, suggesting a homogeneous nature of the preparation (Figure 1C). Further GPC analysis revealed a weight-average molecular weight (Mw) of 73,259 Da for GL-PPQ1 (Figure 1D).

### 3.2. GL-PPQ1 Alleviates Renal I/R-Induced Kidney Pathological Changes

**Figure 2:**
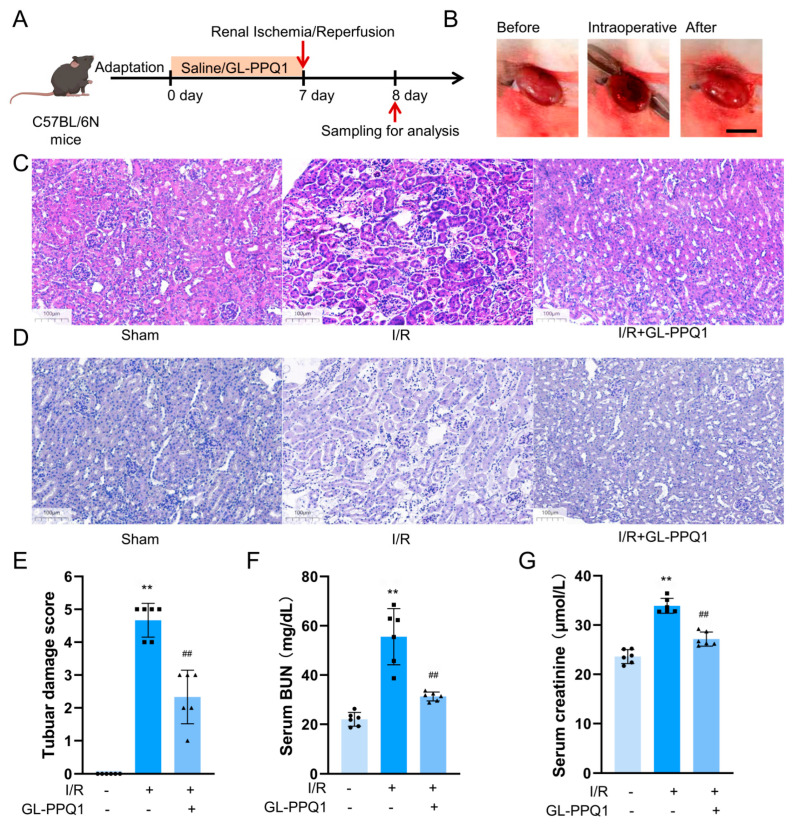
GL-PPQ1 alleviates mouse renal I/R injury. (**A**) Experimental flowchart of renal ischemia–reperfusion. (**B**) The representative pictures of the renal tissue before, during, and after the ischemia–reperfusion procedure. (**C**) Hematoxylin and eosin (H&E) staining. (**D**) PAS staining. (**E**) Renal tubular injury score. (**F**) Blood urea nitrogen levels in serum. (**G**) Serum creatinine levels. (*N* = 6; data are represented as the mean ± SD. ** *p* < 0.01 compared with the sham group. ^##^ *p* < 0.01 compared with the I/R group).

To investigate the protective effect of GL-PPQ1 on renal ischemia/reperfusion (I/R) injury, C57BL/6N mice were pretreated with GL-PPQ1 or saline for 7 days before I/R surgery, and samples were collected 24 h after reperfusion. As shown in Figure 2A, the I/R procedure was successfully established, as evidenced by macroscopic changes in kidney appearance during and after clamping (Figure 2B). Histological analysis using hematoxylin and eosin (HE) staining revealed that kidneys from the I/R group exhibited severe tubular epithelial damage, including tubular dilation, cast formation, and loss of brush border (Figure 2C). In contrast, mice pretreated with GL-PPQ1 showed markedly attenuated histological injury, with renal morphology more comparable to that of the sham group. Additionally, pathological staining (Figure 2D) showed prominent tubular brush border loss and basement membrane damage in the I/R group, reflecting significant structural tubular injury, which was considerably ameliorated following GL-PPQ1 treatment.

Quantitative analysis confirmed these findings. Tubular damage scores were significantly higher in the I/R group compared to sham controls (*p* < 0.01), while GL-PPQ1 treatment significantly reduced the score (*p* < 0.01 vs. I/R, Figure 2E). Consistently, serum levels of blood urea nitrogen (BUN) and creatinine, both markers of renal dysfunction, were markedly increased following I/R injury (*p* < 0.01), but were significantly lowered in the GL-PPQ1-treated group (*p* < 0.01 vs. I/R, Figure 2F,G). Overall, these results demonstrate that GL-PPQ1 effectively alleviates renal tissue damage and preserves kidney function in the context of I/R injury.

### 3.3. GL-PPQ1 Alleviates Renal I/R-Induced Kidney Injury

**Figure 3:**
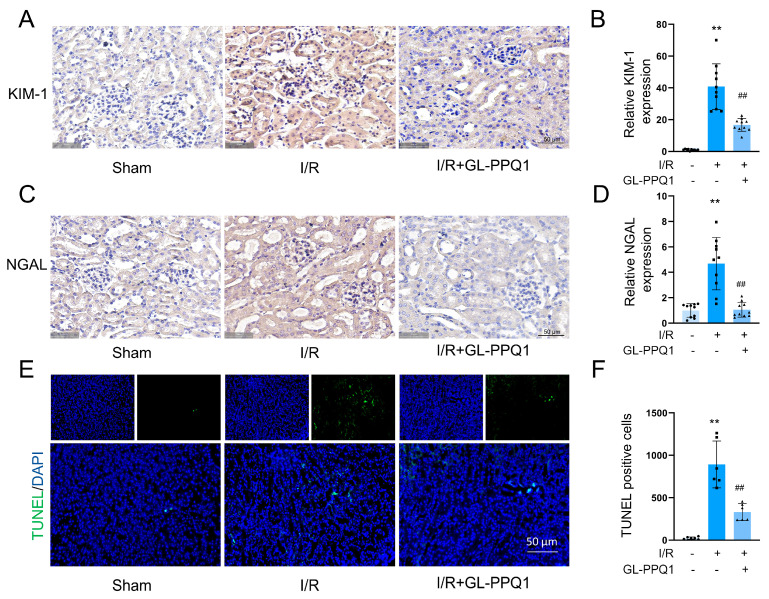
GL-PPQ1 alleviates renal I/R-induced kidney injury. (**A**) Immunohistochemical staining of KIM-1. Scale bar = 50 μm. (**B**) Quantification of KIM-1 expression. (**C**) Immunohistochemical staining for NGAL. Scale bar = 50 μm. (**D**) Quantification of NGAL expression. (**E**) Representative TUNEL staining of the kidney tissue. Scale bar = 50 μm. (**F**) Number of TUNEL-positive cells in the kidney. (*N* = 6; data are represented as mean ± SD. ** *p* < 0.01 compared with the sham group. **^##^** *p* < 0.01 compared with the I/R group).

The evaluation of the protective effect of GL-PPQ1 against renal I/R injury, the expression of two well-established kidney injury biomarkers. Kidney Injury Molecule-1 (KIM-1) and Neutrophil Gelatinase-Associated Lipocalin (NGAL) were assessed by immunohistochemistry. As shown in Figure 3A, KIM-1 expression was markedly elevated in the renal tubular epithelium of the I/R group, indicating severe tubular injury. In contrast, the I/R + GL-PPQ1 group exhibited substantially reduced KIM-1 staining, approaching levels observed in the sham group. Quantitative analysis confirmed this observation, showing significantly increased KIM-1 expression in the I/R group compared to sham (*p* < 0.01), which was significantly attenuated by GL-PPQ1 treatment (*p* < 0.01, Figure 3B).

Similarly, NGAL expression (Figure 3C) was strongly upregulated in the kidneys of I/R mice, reflecting acute renal stress and injury. Treatment with GL-PPQ1 markedly reduced NGAL staining intensity compared to the I/R group. Quantification of NGAL levels revealed a significant increase following I/R injury (*p* < 0.01 vs. sham), which was significantly decreased by GL-PPQ1 pretreatment (*p* < 0.01, Figure 3D). These findings demonstrate that GL-PPQ1 effectively mitigates I/R-induced renal injury by downregulating the expression of key tubular damage markers, further supporting its renoprotective role.

Apoptosis, a form of programmed cell death, is recognized as the predominant mode of cell death during I/R injury induced by oxidative stress [30]. TUNEL staining revealed a marked increase in apoptotic cells within the kidney tissue of the I/R group compared to the sham-operated group. Notably, this I/R-induced increase in apoptosis was significantly attenuated by GL-PPQ1 treatment (Figure 3E,F). These findings demonstrate that GL-PPQ1 effectively alleviates renal tubular cell apoptosis triggered by renal I/R injury.

### 3.4. GL-PPQ1 Alleviates Renal I/R-Induced Oxidative Stress and Inflammation

**Figure 4:**
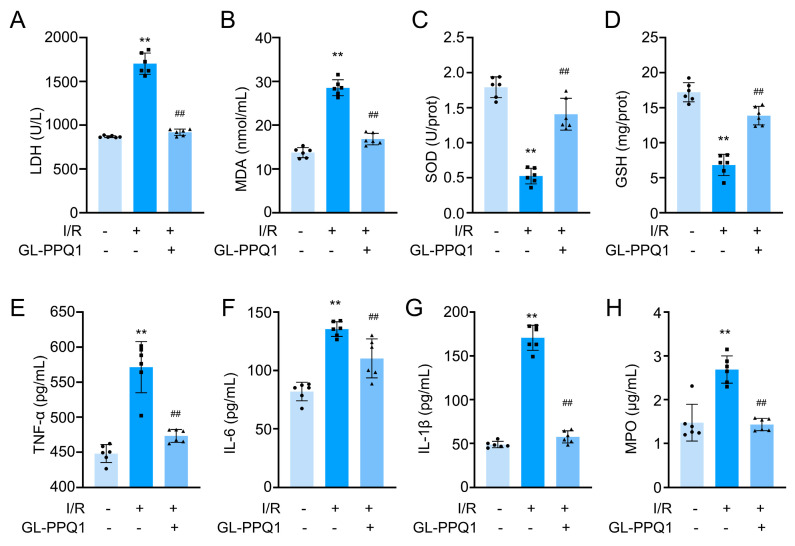
GL-PPQ1 alleviated I/R-induced mice renal injury, oxidative stress, and inflammation in mice. (**A**) Serum LDH level. (**B**) Serum MDA content. (**C**) Serum SOD activity. (**D**) Glutathione Content. (**E**) Serum TNF-α level. (**F**) Serum IL-6 level. (**G**) Serum IL-1β level. (**H**) Serum MPO level. (*N* = 6; data are represented as mean ± SD. ** *p* < 0.01 compared with the sham group. ^##^ *p* < 0.01 compared with the I/R group).

To evaluate the protective effects of GL-PPQ1 on renal I/R injury, we examined biochemical markers of tissue injury, oxidative stress, and inflammation (Figure 4). As shown in Figure 4A,B, the levels of lactate dehydrogenase (LDH) and malondialdehyde (MDA) were significantly increased in the I/R group compared with the sham group (*p* < 0.01), indicating severe cellular damage and lipid peroxidation. Consistent with the TUNEL staining, GL-PPQ1 treatment markedly reduced LDH and MDA levels (*p* < 0.01), suggesting attenuation of renal injury and oxidative damage. Conversely, the activities of antioxidant indicators, including superoxide dismutase (SOD) and glutathione (GSH), were significantly decreased after I/R (*p* < 0.01), whereas GL-PPQ1 administration significantly restored their levels (Figure 4C,D).

Furthermore, the concentrations of pro-inflammatory cytokines tumor necrosis factor-α (TNF-α), interleukin-6 (IL-6), and interleukin-1β (IL-1β) were markedly elevated following I/R (*p* < 0.01), but were significantly suppressed by GL-PPQ1 treatment (Figure 4E–G). Similarly, myeloperoxidase (MPO) activity, a marker of neutrophil infiltration, was strongly increased in the I/R group and effectively reduced by GL-PPQ1 (*p* < 0.01) (Figure 4H). Collectively, these findings demonstrate that GL-PPQ1 alleviates renal I/R-induced tissue injury by reducing oxidative stress and inflammation, while enhancing endogenous antioxidant defense.

### 3.5. Underlying Mechanism of GL-PPQ1 in Alleviating Renal I/R Injury

**Figure 5:**
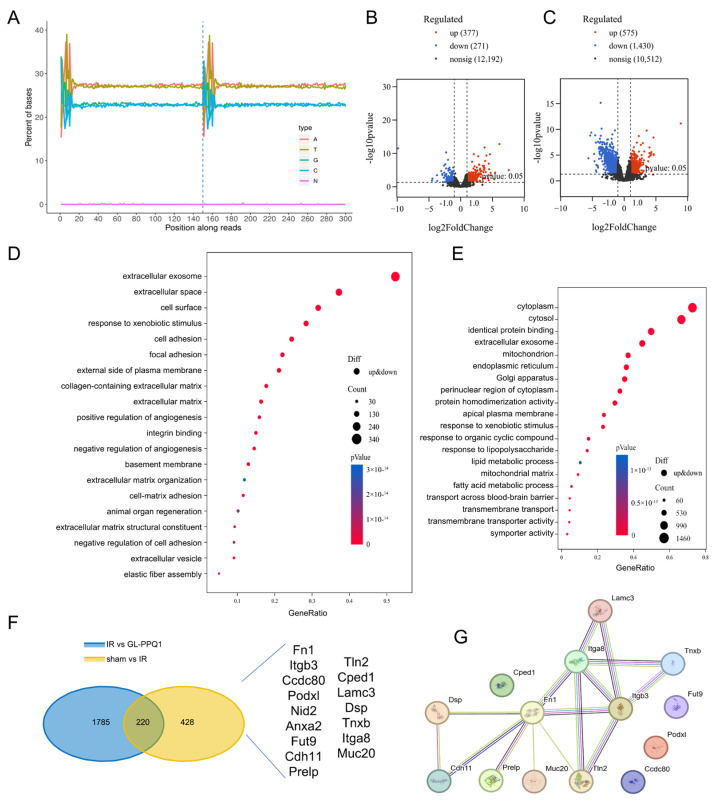
Transcriptomic analysis reveals the underlying mechanism of GL-PPQ1 in mice renal I/R injury. (**A**) Read distribution across transcripts in the sham, I/R, and I/R + GL-PPQ1 groups for RNA-seq quality assessment. (**B**) Volcano plot showing DEGs between I/R and Sham groups. (**C**) Volcano plot of DEGs between I/R + GL-PPQ1 and I/R groups. (**D**) GO enrichment analysis of DEGs between I/R and Sham groups. (**E**) GO enrichment analysis of DEGs between I/R + GL-PPQ1 and I/R groups. (**F**) The overlap of DEGs between I/R vs. GL-PPQ1 and Sham vs. I/R groups. (**G**) PPI network of 220 overlapping DEGs from STRING.

The exploration of the molecular mechanisms underlying the protective effect of GL-PPQ1 against renal I/R injury involved RNA sequencing of kidney tissues from sham, I/R, and I/R + GL-PPQ1-treated mice. As shown in Figure 5A, read distribution across transcripts was consistent across all groups, indicating high-quality and unbiased RNA-seq data suitable for further analysis. Differential gene expression analysis revealed significant transcriptional changes caused by I/R, with 277 genes notably upregulated and 271 downregulated compared to the sham group (Figure 5B). Treatment with GL-PPQ1 resulted in considerable transcriptomic modulation, with 375 genes upregulated and 1049 downregulated relative to the I/R group (Figure 5C).

Gene Ontology (GO) enrichment analysis revealed that I/R injury activated genes associated with extracellular matrix organization, oxidative stress response, and cell adhesion (Figure 5D). In contrast, treatment with GL-PPQ1 enriched gene sets linked to cytoplasmic activity, epithelial structure, lipid metabolism, and oxidative homeostasis (Figure 5E). This suggests that GL-PPQ1 effectively reverses stress-induced transcriptional programs and restores cellular homeostasis.

To investigate the underlying mechanism of GL-PPQ1, a Venn diagram analysis of differentially expressed genes (DEGs) revealed that 220 genes were common between the sham vs. I/R and I/R vs. GL-PPQ1 group comparisons (Figure 5F). Notably, several genes, including Fn1, Itgb3, Podxl, Nid2, Tnxb, Prelp, and Ccdc80, were present in both comparisons, underscoring their potential role in the renoprotective effects of GL-PPQ1. Additionally, a protein–protein interaction (PPI) network analysis (Figure 5G) demonstrated that these genes are part of a closely linked network, primarily related to extracellular matrix remodeling and cellular adhesion. These transcriptomic findings suggest that GL-PPQ1 mitigates renal I/R injury by regulating integrin β3/Fn1 interaction.

### 3.6. Integrin β3/Fn1 Interaction Was Regulated by GL-PPQ1 in Renal I/R Mice

**Figure 6:**
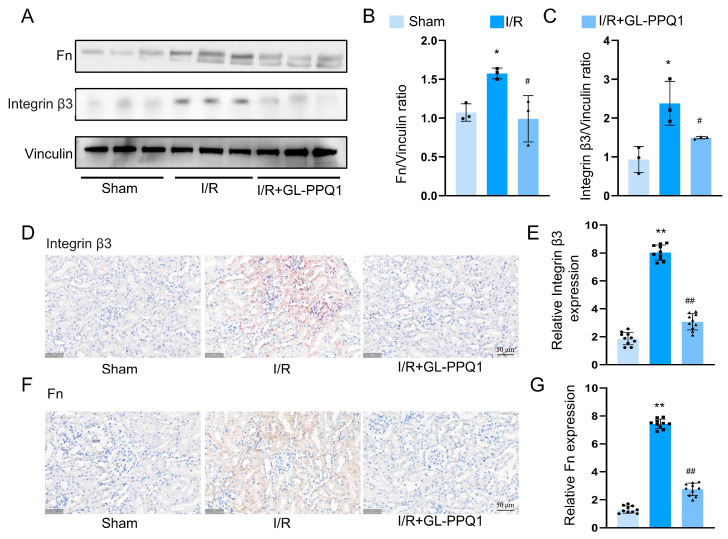
GL-PPQ1 regulates integrin β3 and fibronectin expression in mice renal I/R injury. (**A**) Western blot of Fn, integrin β3, and Vinculin in Sham, I/R, and I/R + GL-PPQ1 groups. (**B**) Fn/Vinculin ratio variation in I/R is reduced by GL-PPQ1. (**C**) Integrin β3/Vinculin Ratio. (**D**) Immunohistochemistry of integrin β3. Scale bar = 50 μm. (**E**) Quantification of integrin β3 IHC. (**F**) Immunohistochemistry of fibronectin. Scale bar = 50 μm. (**G**) Quantification of Fn IHC. (*N* = 3 in Western blot and *N* = 6 in IHC assay; data are shown as mean ± SD. * *p* < 0.05 and ** *p* < 0.01 compared with the sham group; ^#^ *p* < 0.05 and **^##^** *p* < 0.01 compared with the I/R group). Original Western blot images for Figure 6A are included in Supplementary Figures S1–S3.

To determine the regulatory effects of GL-PPQ1 on the interaction between integrin β3 and fibronectin (Fn1) during renal I/R injury, we evaluated their protein levels using Western blot and immunohistochemistry. As shown in Figure 6A, Western blot analysis indicated that I/R significantly increased the protein levels of Fn1 and integrin β3 compared with the sham group. Densitometric analysis revealed a notable increase in the Fn1/Vinculin ratio (Figure 6B) and the integrin β3/Vinculin ratio (Figure 6C) in the I/R group. Importantly, the GL-PPQ1 treatment significantly reduced the elevated expression of both Fn1 and integrin β3, suggesting its role in suppressing their upregulation during I/R injury. Additionally, immunohistochemical staining showed strong positive signals for integrin β3 (Figure 6D) and Fn1 (Figure 6F) in the renal tissues subjected to I/R, while sham kidneys exhibited minimal staining. Quantitative analysis revealed a significant increase in the relative expression of integrin β3 (Figure 6E) and Fn1 (Figure 6G) after I/R, which were significantly reduced following GL-PPQ1 treatment. These findings suggest that renal I/R injury causes excessive expression of integrin β3 and Fn1, while GL-PPQ1 treatment effectively lowers their levels.

### 3.7. GL-PPQ1 Inhibited ROS Production and Cellular Apoptosis in OGD/R-Treated HK2 Cells

**Figure 7:**
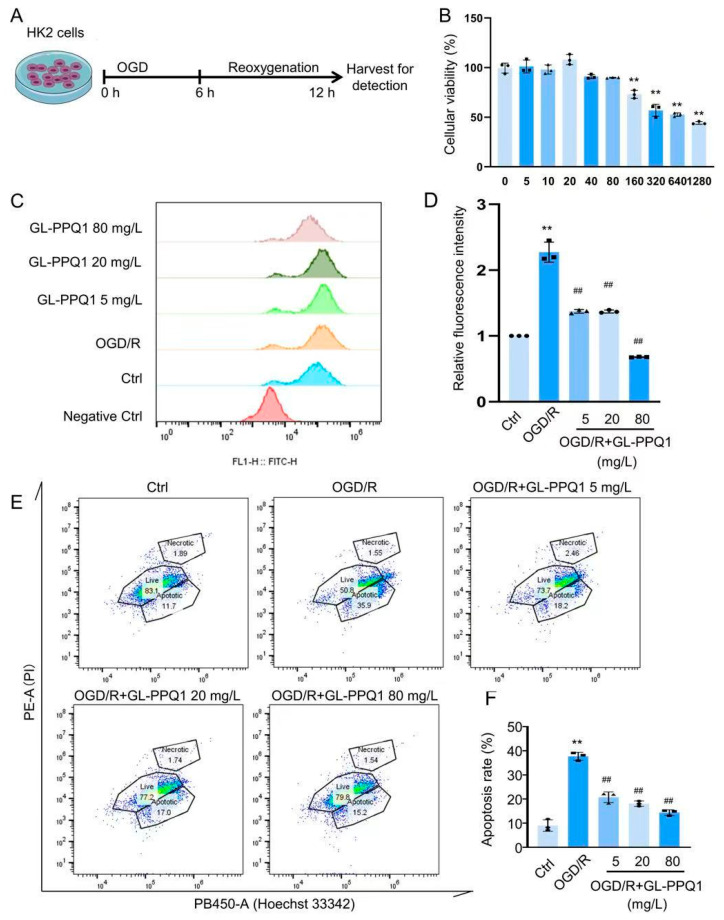
GL-PPQ1 inhibited ROS production and cellular apoptosis in OGD/R-treated HK2 cells. (**A**) The oxygen–glucose deprivation/reoxygenation (OGD/R) model. (**B**) Effect of GL-PPQ1 on HK2 cell viability under OGD/R conditions. (**C**) ROS level. (**D**) Statistical graph of ROS levels. (**E**) Cellular apoptosis. (**F**) Statistical graph of cellular apoptosis rates. (*N* = 3; data are shown as mean ± SD. ** *p* < 0.01 compared with the sham group; **^##^** *p* < 0.01 compared with the I/R group).

The in vitro model of I/R damage was created by the HK2 cells, which underwent 6 h of oxygen–glucose deprivation (OGD) followed by 12 h of reoxygenation (Figure 7A). To determine the appropriate concentration range of GL-PPQ1, cell viability was assessed using various doses (0–1280 mg/L) under OGD/R conditions. As shown in Figure 7B, GL-PPQ1 at concentrations of 5–80 mg/L had no significant cytotoxic effect, whereas higher concentrations (≥80 mg/L) markedly decreased cell viability (*p* < 0.01) in a dose-dependent manner. These findings indicate that low concentrations of GL-PPQ1 are safe for HK2 cells, providing a suitable range for subsequent experiments investigating its protective effects against OGD/R-induced oxidative stress and apoptosis. Thus, 5, 20, and 80 mg/L were chosen to evaluate the anti-oxidative stress activity. The results showed GL-PPQ1 (5, 20, and 80 mg/L) significantly inhibited ROS level (Figure 7C,D). Additionally, GL-PPQ1 markedly suppressed OGD/R-induced cellular apoptosis (Figure 7E,F). The above results indicate GL-PPQ1 has a renoprotective effect via inhibiting oxidative stress and cellular apoptosis.

### 3.8. GL-PPQ1 Inhibited Integrin β3/Fn1 Expression in OGD/R-Treated HK2 Cells

**Figure 8:**
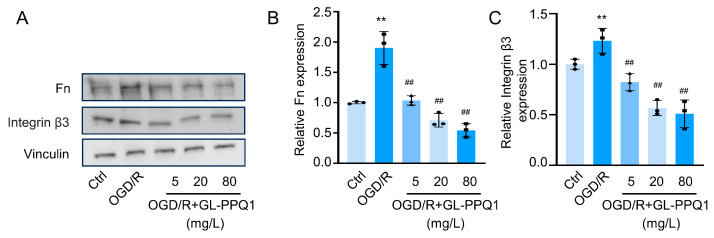
GL-PPQ1 attenuates OGD/R-induced upregulation of integrin β3 and fibronectin in HK2 cells. (**A**) Fn1 and integrin β3 expression in control, OGD/R, and GL-PPQ1-treated cells. (**B**) Quantification of Fn1 expression normalized to Vinculin. (**C**) Quantification of integrin β3 expression normalized to Vinculin. (*N* = 3; data are shown as mean ± SD. ** *p* < 0.01 compared with the sham group; ^##^ *p* < 0.01 compared with the I/R group). Original Western blot images for Figure 8A are included in Supplementary Figures S4–S8.

The data presented in Figure 8 demonstrate that GL-PPQ1 exerts a suppressive effect on the expression of Fn1 and integrin β3 in HK2 cells exposed to OGD/R, a model mimicking ischemic injury. Western blot analysis demonstrated that OGD/R treatment markedly increased the protein expression of Fn1 and integrin β3 in HK2 cells compared to the control group. Upon administration of GL-PPQ1 at concentrations of 5, 20, and 80 mg/L, a dose-dependent suppression of both proteins was observed, as shown in Figure 8A. Quantitative analysis of Fn expression (Figure 8B) revealed a significant elevation under OGD/R conditions, reaching approximately 2.2-fold relative to control, which was progressively reduced to ~1.8, ~1.4, and ~1.1-fold with increasing GL-PPQ1 concentrations. Similarly, Figure 7C shows that integrin β3 expression rose to ~2.0-fold following OGD/R, and was attenuated to ~1.7, ~1.3, and ~1.0-fold upon GL-PPQ1 treatment. These findings indicate that GL-PPQ1 effectively inhibits OGD/R-induced upregulation of extracellular matrix (Fn1) and cell adhesion (integrin β3) proteins, suggesting its potential role in mitigating ischemia-associated cellular remodeling and adhesion signaling.

## 4. Discussions

In this study, the extraction and purification process were performed. The yield of GL-PPQ1 (0.16% *w*/*w*) was comparable to other bioactive GLPP fractions obtained by similar membrane separation and ethanol precipitation methods, where yields of 0.1–0.5% have been reported by Luo et al. (2024) [16]. Despite appearing modest, this yield is supported by the scalability of the AKTAflux6 hollow fiber membrane system and by the high biological potency of GL-PPQ1 at a low dose of 100 mg/kg [16]. This study provides compelling evidence that GL-PPQ1 exerts significant protective effects against renal I/R injury, both in vivo and in vitro, by modulating oxidative stress, inflammation, and extracellular matrix remodeling. Using a murine I/R model and an OGD/R-induced HK2 cell injury model, we demonstrate that GL-PPQ1 not only alleviates histopathological damage and renal dysfunction but also attenuates the expression of key injury markers and adhesion molecules implicated in renal fibrosis and maladaptive repair.

In the murine I/R model, GL-PPQ1 pretreatment significantly reduced tubular damage, as shown by improved histological structure, lower injury scores, and decreased serum BUN and creatinine levels. These results align with those of Hou et al. (2023) [31], who showed that antioxidant-based interventions, like SOD2 mRNA delivery, can effectively lessen I/R-induced renal dysfunction by maintaining tissue integrity and reducing oxidative damage. Additionally, similar kidney protective effects have been reported for *G. lucidum* polysaccharide peptides (GLPPs), notably lowered tubular necrosis and enhanced renal function in I/R-injured rats by scavenging reactive oxygen species [31,32].

The observed reduction in KIM-1 and NGAL expression further supports the renoprotective effect of GL-PPQ1, as these markers are widely recognized indicators of acute tubular injury. Oxidative stress and inflammation [31,32] are central to the pathogenesis of I/R injury. Our data show that GL-PPQ1 significantly decreased MDA and LDH levels while restoring antioxidant defenses such as SOD and GSH. These effects were accompanied by a marked reduction in pro-inflammatory cytokines (TNF-α, IL-6, IL-1β) and MPO activity, indicating suppression of neutrophil infiltration and systemic inflammation. These results are similar to the review by Asgharpour et al. (2021), which highlighted the efficacy of herbal antioxidants in reducing oxidative and inflammatory responses in renal I/R models [33].

Transcriptomic analysis provided further mechanistic insight, revealing that GL-PPQ1 reversed I/R-induced changes in gene expression, particularly those related to extracellular matrix organization and cell adhesion. Notably, Fn1 and Itgb3 were among the key differentially expressed genes downregulated by GL-PPQ1. These genes are known to mediate integrin signaling and matrix accumulation, processes that contribute to post-ischemic fibrosis and maladaptive repair. Li et al. (2021) demonstrated that integrin β3 promotes tubular cell senescence and fibrosis in renal injury models [34], while Wang et al. (2024) showed that Fn1+ macrophage recruitment via integrin-rich vesicles exacerbates I/R-induced inflammation [35]. Protein-level validation confirmed that GL-PPQ1 significantly suppressed the expression of fibronectin and integrin β3 in both renal tissues and OGD/R-treated HK2 cells, supporting its role in modulating ECM remodeling and adhesion signaling.

The in vitro data further support a direct cellular effect of GL-PPQ1. Under OGD/R conditions, HK2 cells exhibited elevated Fn1 and integrin β3 expression, mimicking the fibrotic response observed in vivo. GL-PPQ1 treatment led to a dose-dependent reduction in these proteins, suggesting interference with integrin-mediated adhesion and matrix remodeling pathways. This is consistent with the broader understanding of integrin ECM interactions as therapeutic targets in renal injury, as discussed by Li et al. (2024), who emphasized the role of regulated cell death and adhesion signaling in I/R-induced acute kidney injury [36].

Taken together, our data support the hypothesis that GL-PPQ1 protects against I/R-induced AKI by attenuating oxidative stress and modulating integrin β3/Fn1-mediated extracellular matrix remodeling. These findings highlight the therapeutic potential of *Ganoderma lucidum*-derived polysaccharide peptides in managing AKI and possibly other forms of renal injury and support their development as dietary supplements for renal protection. While GL-PPQ1 was not structurally characterized in this study, it is hypothesized to possess a similar galactoglucan backbone as GL-PPSQ2 and GL-PPQ3, as were previously reported by Luo et al. (2025) [11] and Lin et al. (2023) [10]. This study has some limitations. First, only male mice were used, and sex-based differences in renal I/R injury responses were not explored. Second, a single dose of GL-PPQ1 (100 mg/kg) was tested in vivo; future dose–response studies are warranted. Third, the short reperfusion period (24 h) captures acute injury but does not reflect long-term outcomes. Finally, the precise molecular binding mechanism between GL-PPQ1 and integrin β3/knockdown and Fn1 inhibition approaches requires further investigation. The larger sample sizes and formal power calculations will be applied in future studies to validate these findings further. The mRNA-level validation by qPCR was not performed; this is a limitation of the study that will be addressed in future studies.

## 5. Conclusions

This study demonstrates that GL-PPQ1, a novel polysaccharide peptide derived from *Ganoderma lucidum*, exerts significant renoprotective effects against ischemia–reperfusion-induced acute kidney injury through multiple mechanisms. In both the murine I/R model and OGD/R-treated HK-2 cells, GL-PPQ1 effectively reduced tubular structural damage, preserved renal function, and attenuated oxidative stress and inflammatory responses. Transcriptomic analysis combined with protein-level validation identified the integrin β3/Fn1 signaling axis as a key mechanistic target, with GL-PPQ1 significantly suppressing the expression of integrin β3 and fibronectin 1 in renal tissues and tubular epithelial cells, thereby mitigating extracellular matrix remodeling and maladaptive adhesion signaling associated with post-ischemic kidney injury. These findings highlight GL-PPQ1 as a promising candidate for further investigation in AKI prevention and management. Future studies involving functional pathway validation, dose–response optimization, and long-term safety assessment are necessary to fully establish its translational potential.

## Data Availability

The original contributions presented in the study are included in the article/Supplementary Material; further inquiries can be directed to the corresponding authors.

## Supplementary Materials

The following supporting information can be downloaded at https://www.mdpi.com/article/10.3390/biom16040610/s1, Table S1: Antibody used, Figures S1–S3: Original Western blots images for Figure 6A; Figures S4–S8: Original Western blots images for Figure 8A.
