# Supplementary material for: Polysaccharide Peptide from Ganoderma lucidum Reduces Acute Kidney Injury Through Regulating the Integrin β3/Fn1 Axis
Source: Biomolecules. 2026 Apr 20;16(4):610. doi: 10.3390/biom16040610 (PMC13115497; doi:10.3390/biom16040610)
Supplement: Supplementary file 1 [file biomolecules-16-00610-s001.zip › biomolecules-4231032-Supplement material-r1-updated/biomolecules-4231032-Supplement material-r1-updated-figure.pdf]

biomolecules

MDPI

Some of the bands were not displayed by the markers due to high protein exposure signals. The protein and molecular weight are labeled on the graph.

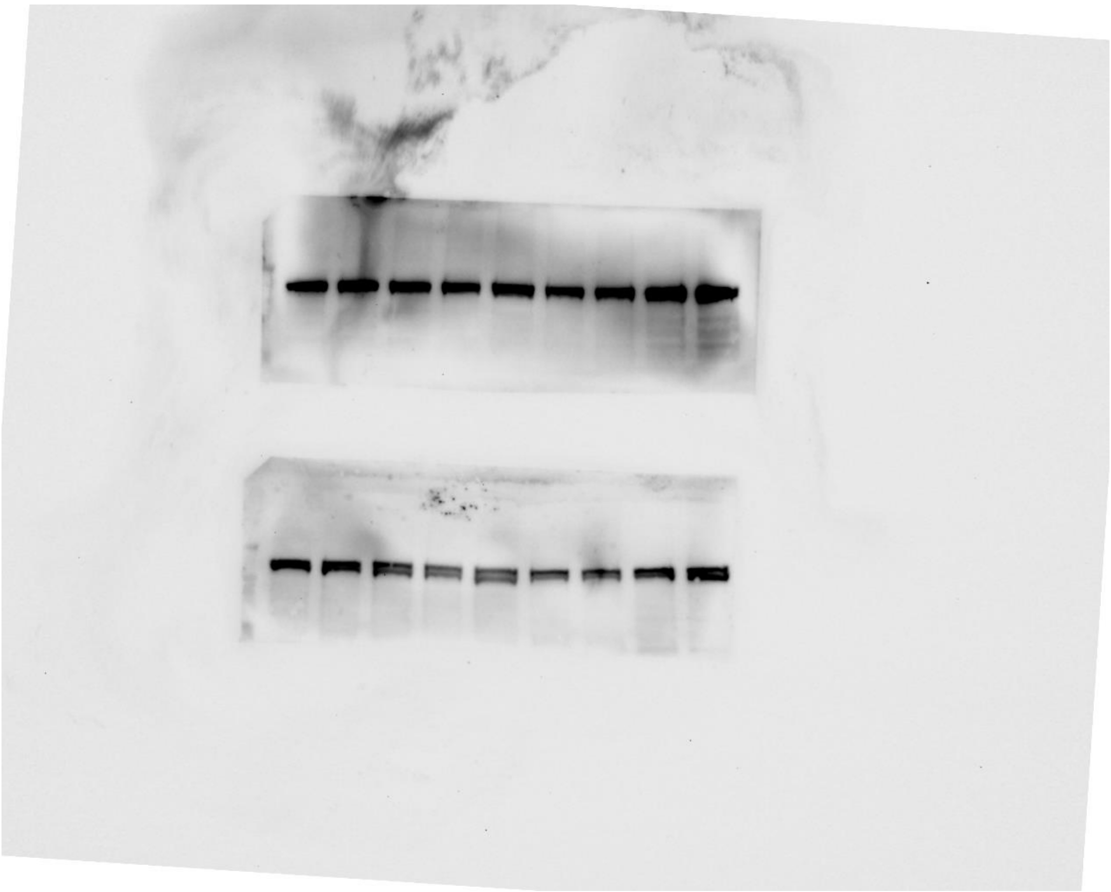

Figure S1. Fig 6: Vinculin.

 https://doi.org/10.3390/xxxxx

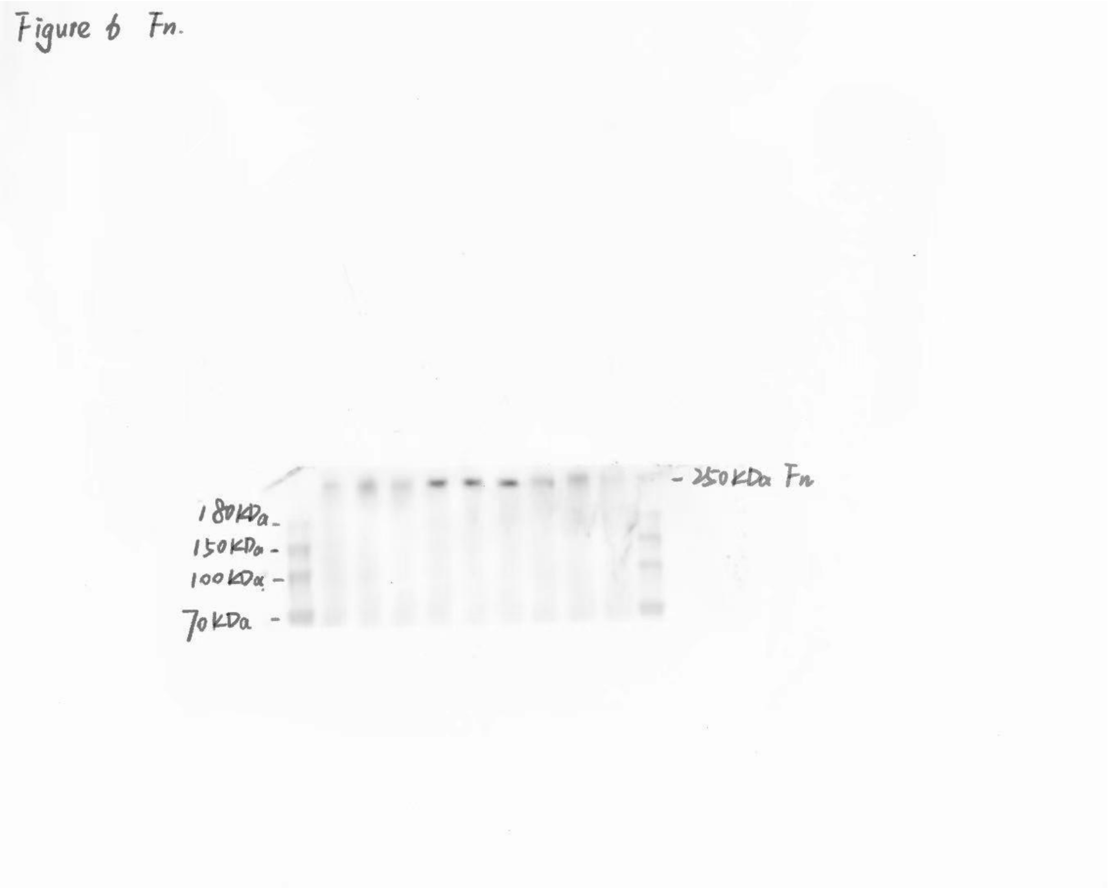

Figure S2. Fig 6. Fn.

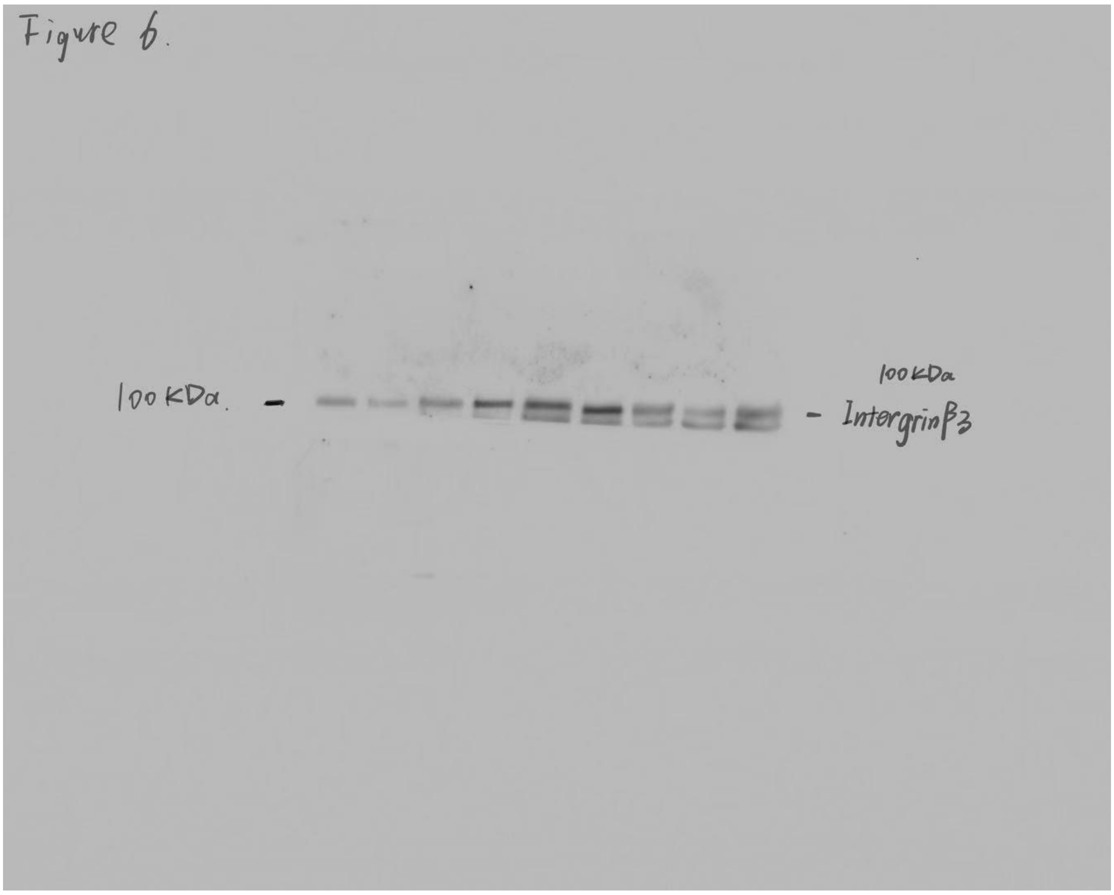

Figure S3. Fig 6. Integrin β3.

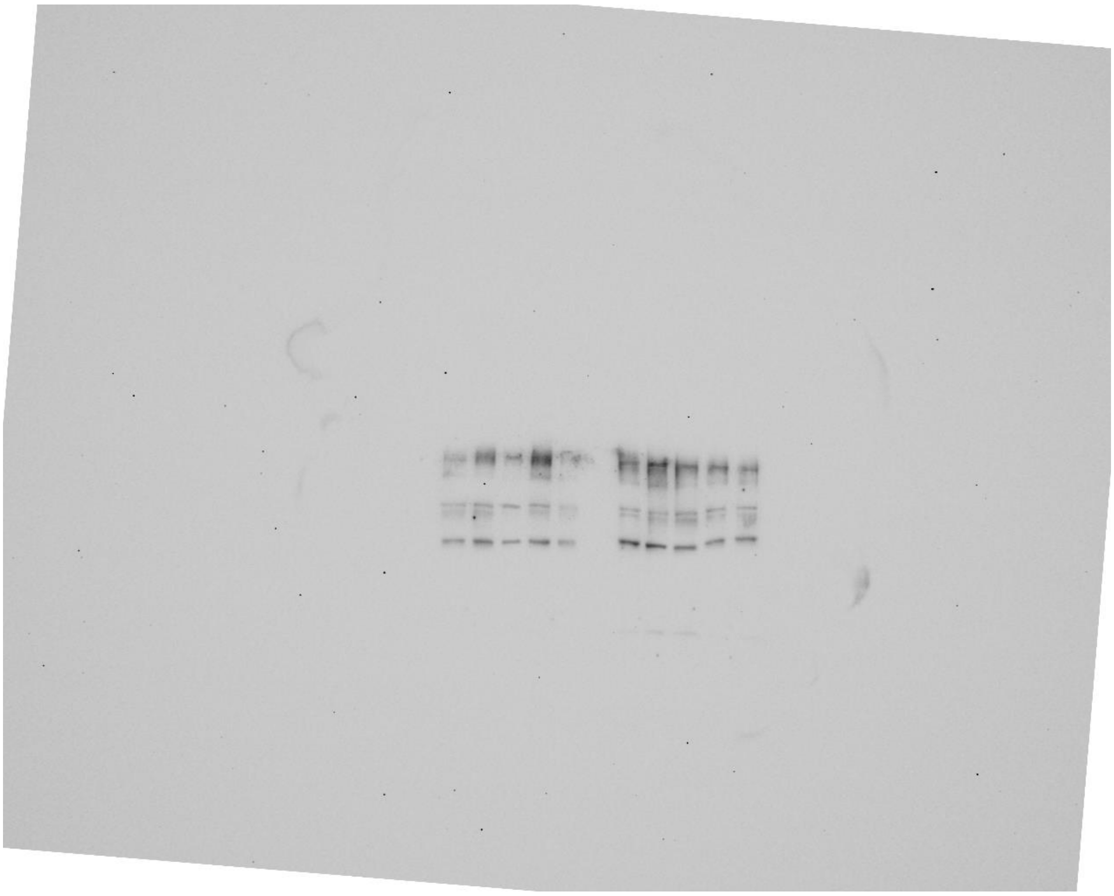

Figure S4. Fig. 8. FN Vinculin CD 61.

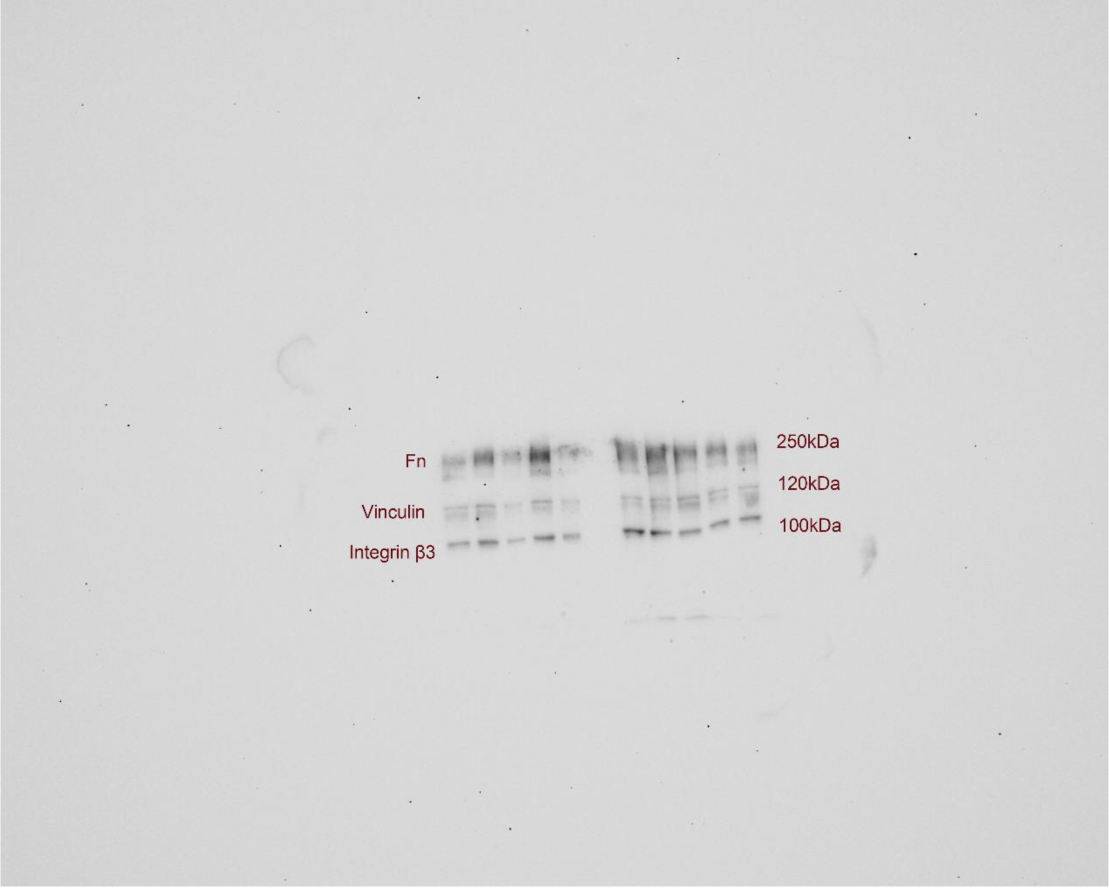

Figure S5. Fig. 8-1 integrin β3/Fn1.

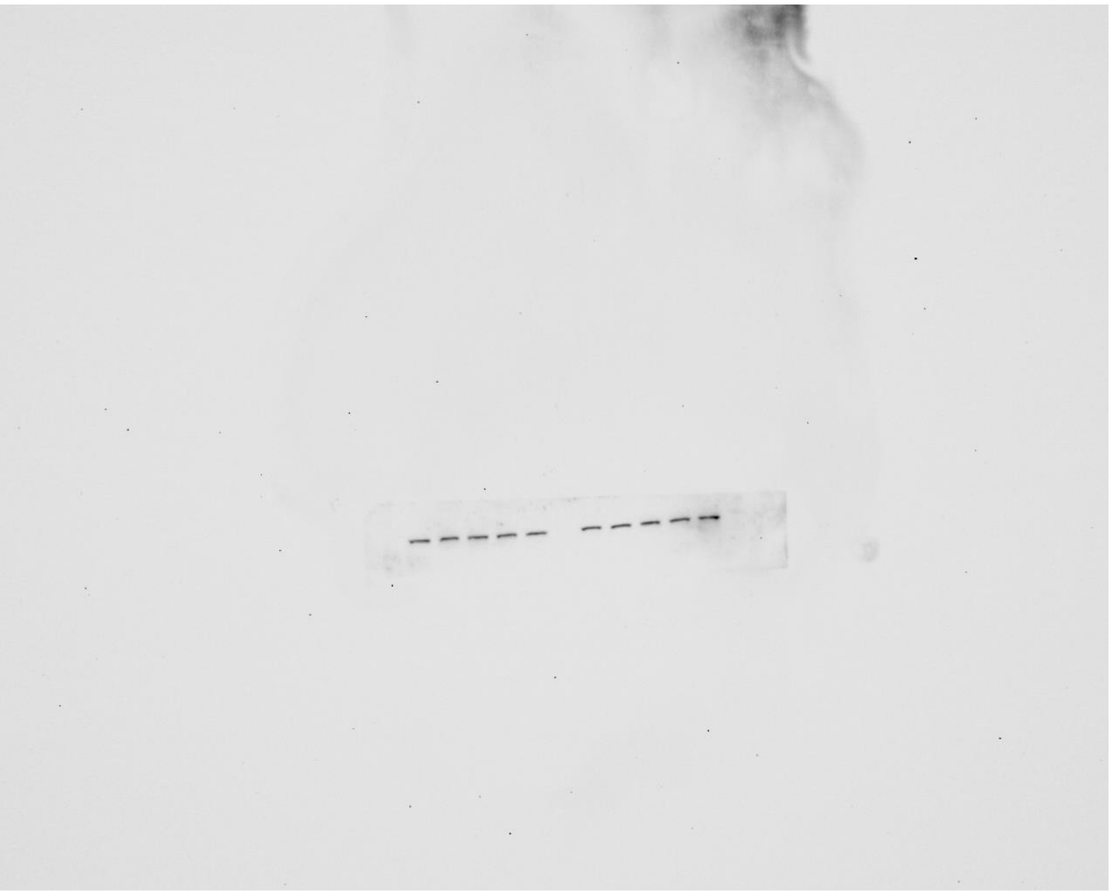

Figure S6. Fig. 8-2 Vinculin.

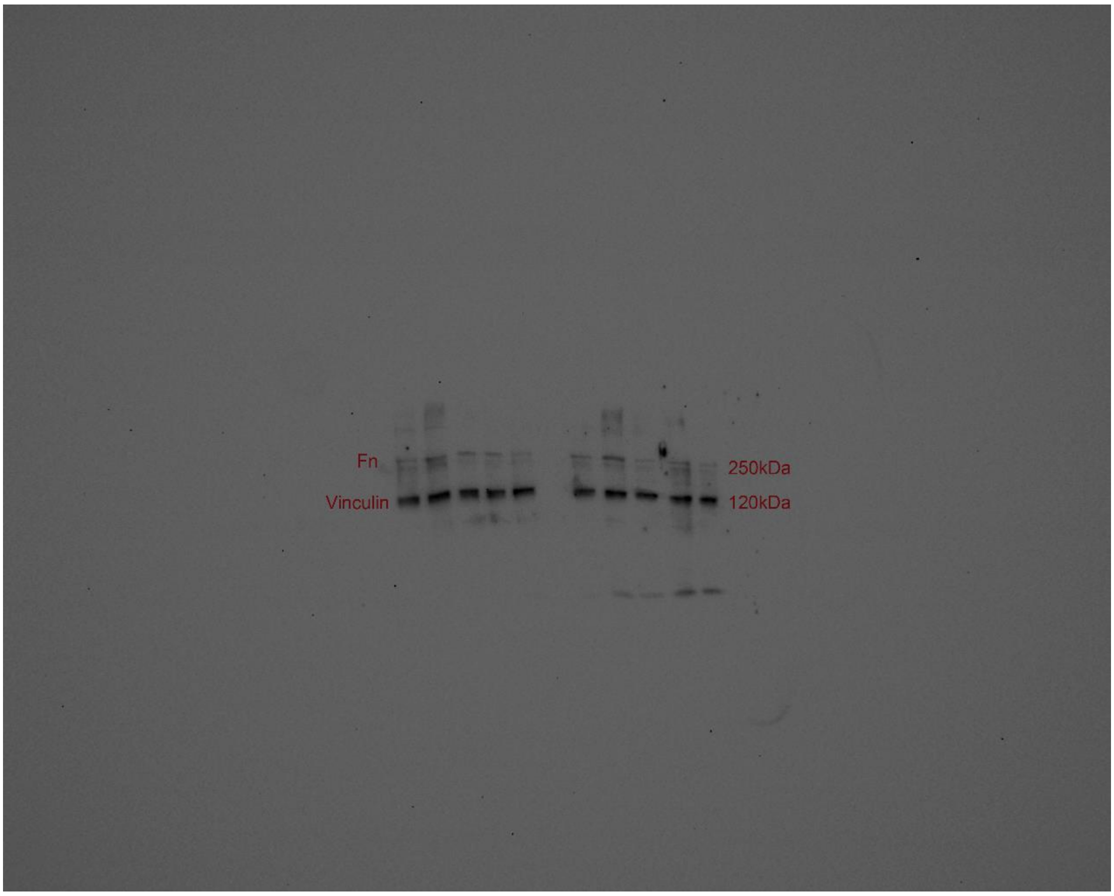

Figure S7. Fig. 8-3 Fn/Vinculin.

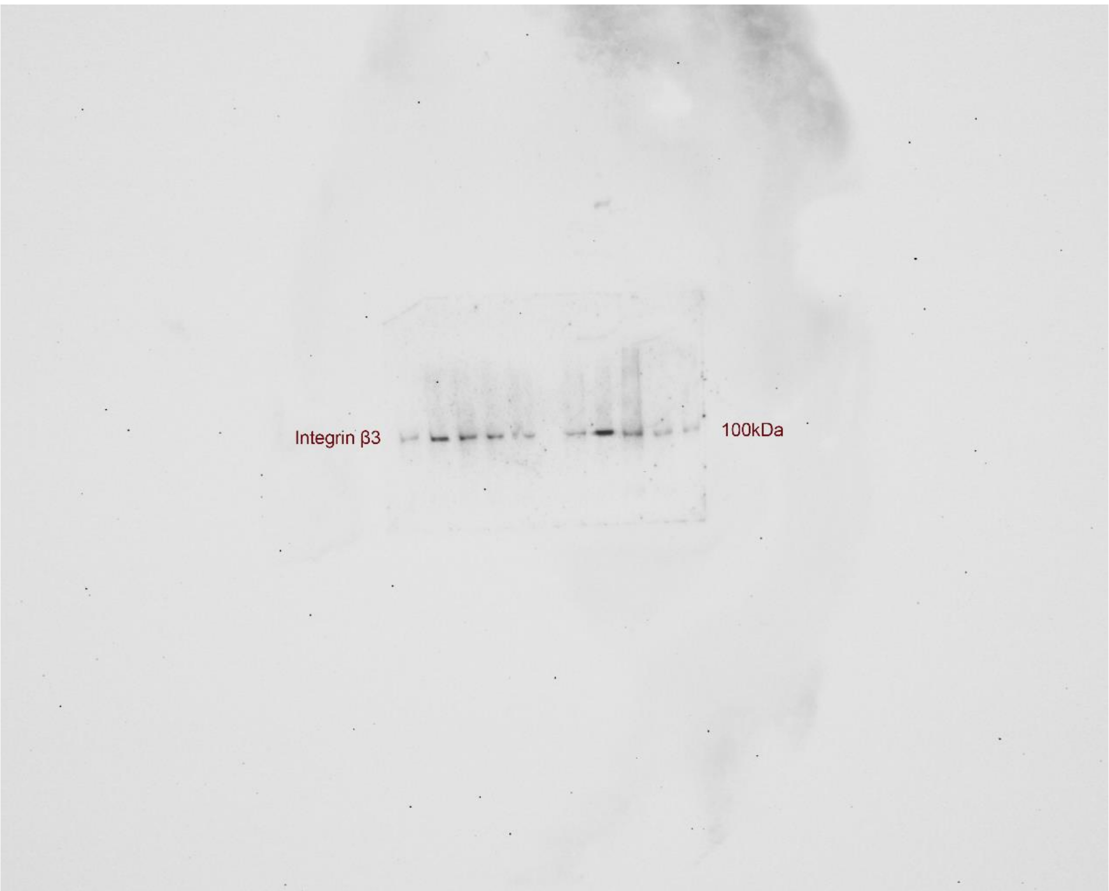

Figure S8. Fig. 8 integrin β3.
