# Supplementary material for: Polysaccharide Peptide from Ganoderma lucidum Reduces Acute Kidney Injury Through Regulating the Integrin β3/Fn1 Axis
Source: Biomolecules. 2026 Apr 20;16(4):610. doi: 10.3390/biom16040610 (PMC13115497; doi:10.3390/biom16040610)
Supplement: Supplementary file 1 [file biomolecules-16-00610-s001.zip › biomolecules-4231032-Supplement material-r1-updated/biomolecules-4231032-supplementary-r1-table.pdf]

Table S1 Antibody used

Name	Manufacturer	Item number	Dilution ratio	Host/subtype
KIM-1/HAVCR1 Polyclonal antibody	Proteintech	30948-1-AP	1:1000	Rabbit/IgG
Vinculin Polyclonal antibody	Proteintech	26520-1-AP	1:5000	Rabbit/IgG
Fibronectin Monoclonal antibody	Proteintech	66042-1-Ig	1:2000	Mouse/IgG1
CD61 / Integrin beta 3 Monoclonal antibody	Proteintech	66952-1-Ig	1:2000	Mouse/IgG2b
Multi-rAb® HRP-Goat Anti-Rabbit Recombinant Secondary Antibody (H+L)	Proteintech	RGAR001	1:10000	Goat
Multi-rAb® HRP-Goat Anti-Mouse Recombinant Secondary Antibody (H+L)	Proteintech	RGAM001	1:10000	Goat
